# MRI-guided intracerebral convection-enhanced injection of gliotoxins to induce focal demyelination in swine

**DOI:** 10.1371/journal.pone.0204650

**Published:** 2018-10-01

**Authors:** Lukasz Kalkowski, Izabela Malysz-Cymborska, Dominika Golubczyk, Miroslaw Janowski, Piotr Holak, Kamila Milewska, Dorota Kedziorek, Zbigniew Adamiak, Wojciech Maksymowicz, Piotr Walczak

**Affiliations:** 1 Dept of Neurology and Neurosurgery, Faculty of Medical Sciences, University of Warmia and Mazury, Olsztyn, Poland; 2 NeuroRepair Dept, Mossakowski Medical Research Centre, Polish Academy of Sciences, Warsaw, Poland; 3 Institute for Cell Engineering, Cellular Imaging Section, The Johns Hopkins University School of Medicine, Baltimore, United States of America; 4 Division Russell H. Morgan Dept. of Radiology and Radiological Science, The Johns Hopkins University School of Medicine, Baltimore, MD, United States of America; 5 Dept of Surgery and Radiology, Faculty of Veterinary Medicine, University of Warmia and Mazury, Olsztyn, Poland; Instituto Cajal-CSIC, SPAIN

## Abstract

Demyelinating disorders such as multiple sclerosis (MS) or transverse myelitis are devastating neurological conditions with no effective cure. Prevention of myelin loss or restoration of myelin are key for successful therapy. To investigate the disease and develop cures animal models with good clinical relevance are essential. The goal of the current study was to establish a model of focal demyelination in the brain of domestic pig using MRI-guided gliotoxin delivery. The rationale for developing a new myelin disease model in the domestic pig was based on the fact that the brain in pigs is anatomically and histologically much more similar to that of humans compared to the rodent brain. For MRI-assisted gliotoxin injection, eight 30 kg pigs were subjected to treatment with lysolecithin (20, 30 mg/ml); or with ethidium bromide (0.0125, 0.05, 0.2 mg/ml). Animals were placed in an MRI scanner for intraparenchymal targeting of gliotoxin into the corona radiata (250 μl over 1h), with real-time monitoring of toxin distribution on T1 scans and monitoring of lesion evolution over seven days using both T1 and T2 scans. After the last MRI, animals were transcardially perfused and brains were processed for histological and immunofluorescent analysis. Gadolinium-enhanced T1 MRI during injection demonstrated biodistribution of the contrast (as a surrogate marker for toxin distribution) and its diffusion through the brain parenchyma. Lesion induction was confirmed on T2-weighted MRI and histopathology, thus enabling the establishment of optimal doses of gliotoxins. To conclude, MRI-guided focal demyelination in swine is accurate and provides real-time confirmation of gliotoxin, thus facilitating placement of focal lesions with high precision. This new model of focal demyelination can be used for further investigation and development of novel therapeutic approaches.

## Introduction

Demyelinating disorders of the central nervous system such as Multiple sclerosis (MS) [1] or transverse myelitis [2] are devastating conditions without effective cure. One of the hallmark features of these disorders are demyelinating lesions. The distribution of demyelinating lesions in MS is variable, with frequent involvement of the optic nerves, spinal cord, periventricular white matter, brainstem, and cerebellum [3]. Therapies capable of restoring the white matter damage and eliminating long-term consequences of relapses are still missing. Cell transplantation-based therapies such as with myelinating glial-restricted precursors were shown to be effective in repairing white matter in small animal models of transverse myelitis [4] and dysmyelination [5]. Cell transplants were also shown to exert immunomodulatory effect and improve neurological deficits in mouse experimental autoimmune/autoallergic encephalomyelitis (EAE) model [6]. EAE, which currently is the most frequently used MS model, owing to clinically relevant pathomechanism of demyelination, has several significant disadvantages, including insufficient cerebral localization of the lesions (where most clinical lesions occur), and, unpredictable and variable localization of lesions, which complicates studies that involve direct targeting of therapeutic agents. Consequently, there is much interest in developing focal models of demyelinating diseases where lesions can be localized, on demand, within a desired target structure, with good control of lesion size. Focal models are typically induced by intraparenchymal injections of gliotoxins, resulting in demyelination [4, 7]. Various toxins have been used to induce and model focal demyelination, including lysolecithin (lysophosphatidylcholine, LPC; [8]) and ethidium bromide (EtBr) [9].

To date, the vast majority of myelin disease modeling has been performed in small animals[4, 10]. While rodents are invaluable tools for studying human disease, due to substantial differences between the rodent and human brain anatomy, modeling neurological diseases in mice is often inaccurate [8]. Cerebral white matter is the best example of these differences, as the brain in mice comprises only 10% of white matter compared to 50% in humans. Additionally, the re-myelination of gliotoxins-induced lesions is very robust in small animal models, which makes testing of therapeutic effects of cell transplantation difficult. There is now consensus in the field of regenerative medicine that prior to clinical translation, therapies should be tested in large animal model anticipating improved clinical relevance in terms of anatomical and pathophysiological features.

Large animal model of focal demyelination is currently not available thus, in this study we focused on developing such model in domestic swine. Swine brain has white matter/gray matter content comparable to that of humans and as such seems highly relevant and appropriate to model MS.

Convection-enhanced delivery (CED) is a technique that has been developed to facilitate and improve the efficacy of targeted injection into the brain parenchyma. CED has been performed with various biochemical compounds in several preclinical models [11, 12], as well as in brain tumor patients [13]. The low velocity of injection in CED minimizes potential damage [14] and results in uniform distribution of active ingredients during intraparenchymal injections [15]. Bankiewicz et al. also contributed an important advancement that further improved the precision of intracerebral injection by performing CED under the guidance of real-time MRI [16, 17]. Performing CED inside the MRI scanner using an MRI contrast agent enables real-time monitoring of infusate distribution [18]. Overall, this method provides excellent control over intraparenchymal cerebral injection, which seems to be ideally suited for inducing focal demyelinating lesions within the white matter of the swine brain.

The overarching goal of our work was to test feasibility of induction of MS-like white matter damage in large animal and to establish more clinically relevant model for translational research.

## Materials and methods

### Animals

All animal procedures were approved by the University of Warmia and Mazury ethics committee and were performed according to ARRIVE guidelines. Eight juvenile, white domestic female pigs, with an average weight of 30 kg were used. Pigs had access to water and food *ad libitum*.

### Gliotoxin preparation

Ethidium bromide solution (EtBr; Sigma, St. Louis, MO, USA) and lysolecithin powder (LPC; Sigma) were diluted in phosphate buffered saline (PBS) at the final concentrations of 0.2, 0.05 and 0.0125 mg/ml for EtBr and 20 and 30 mg/ml for LPC. All gliotoxin solutions were supplemented with MRI contrast agent, gadoteridol (ProHance, Bracco Imaging, Princeton, NJ, USA) at a final concentration of 2 mM.

### Mounting of injection device

**Fig 1:**
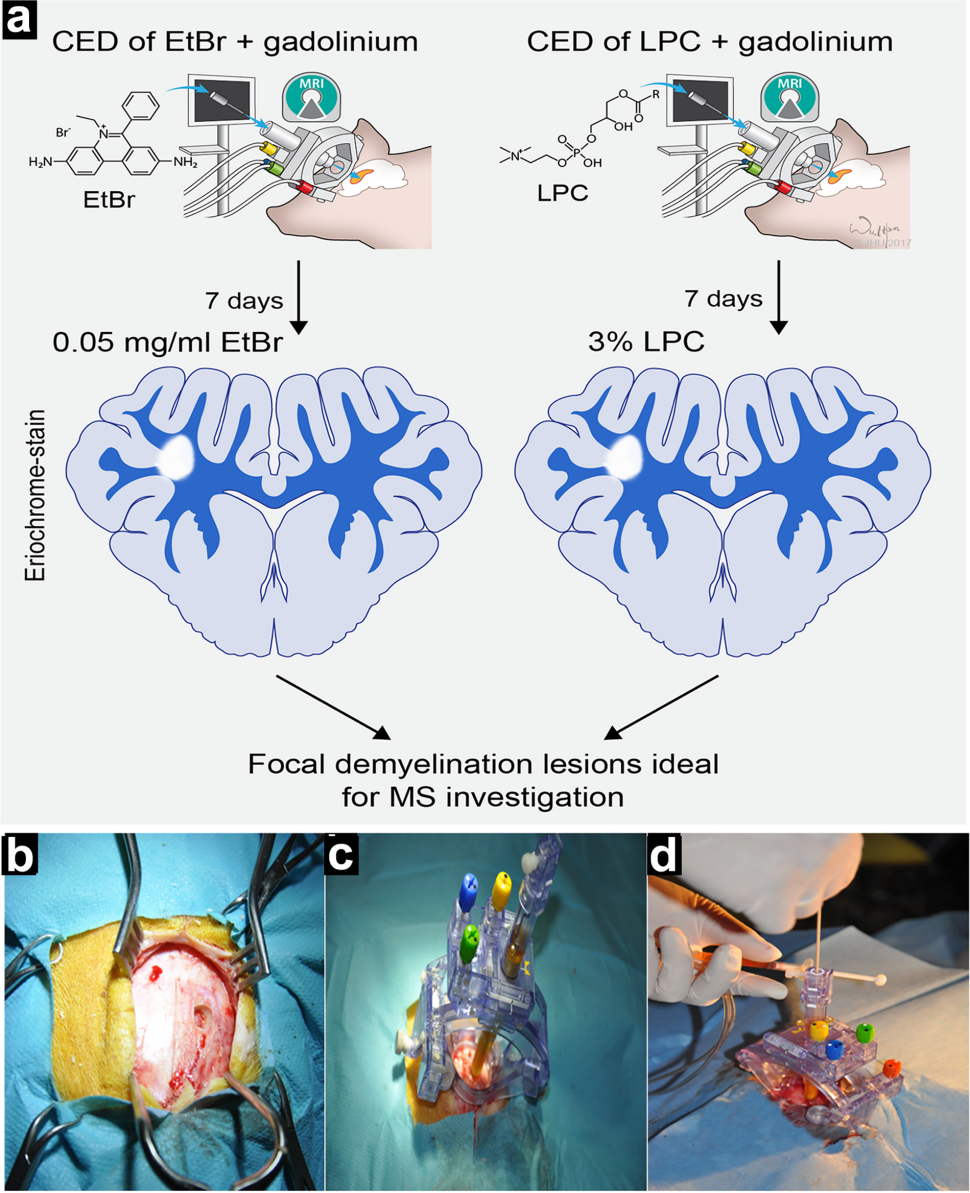
(a) Overall experimental design. (b-d) Photographic images from surgery: directly after burr hole drilling (b), with fixed trajectory device (c) and during cannula placement (d).

Pigs were pre-anesthetized with a subdermal injection of atropine (0.05 mg/kg) and intramuscular injection of ketamine-xylazine solution (6mg/kg and 3 mg/kg, respectively). Anesthesia was maintained with a combination of sevoflurane (1–1,5%) and propofol (3–5 mg/kg/h). During anesthesia, vital parameters were monitored. A midline incision was made in the skin and a 3 mm burr hole was placed unilaterally at the level of the bregma, approximately 11 mm laterally from the midline (**Fig 1A**). The ClearPoint SmartFrame Device (MRI Interventions, Irvine, CA, USA) was fixed on the skull with titanium screws (**Fig 1B**). The SmartFlow catheter (MRI Interventions) was filled with gliotoxin and mounted in a SmartFrame (**Fig 1C**). Animals were placed in a 3 T MRI scanner (Siemens AG, Munich, Germany) and then the cannula trajectory, based on the MRI scan, was adjusted. After calculating the distance to the corona radiata, the cannula was slowly advanced and secured in place. Gliotoxin solution was infused using a CED protocol [19] at a rate of 250 μl/h. During the one-hour infusion of the total 250 μl gliotoxin, T1 images were serially acquired to assess gliotoxin biodistribution in real-time. In two animals, the placement of the cannula was corrected due to suboptimal biodistribution, as visualized on real-time MRI. After injection was completed, the ClearPoint device was removed from the skull and animal was awaken.

### Magnetic resonance imaging

MRI was performed prior to, during the injection, after injection, 1–3 days after injury 7 days after injury. During imaging, animals were anesthetized and positioned prone in a 3T MRI scanner (Magnetom Trio, Siemens AG). A HASTE sequence was used to visualize and adjust the trajectory of the injection cannula to target the corona radiata. The MRI protocol included T2 (TR/TE = 6440/83 ms) and T1 prior and post contrast (TR/TE = 1900/2.5 ms) [20].

### Calculation of the accuracy of injury induction

For evaluation of the dynamic T1+gadolinium imaging during CED procedure as a predictor of the brain injury area we used a method previously described by us for intra-arterial targeting [21]. We segmented and measured the area of the contrast enhancement (cm^2^) for four brain regions: 1) the overlapping area for both T1 enhancement and T2 hyperintensity (defined as true positive; TP); 2) the T1 enhancement area which did not overlap with T2 hyperintensity (false positive; FP); 3) the area of T2 hyperintensity, which was enhanced on T1 MRI (false negative; FN); and 4) the area of the brain with no signal changes on T1 or T2 (true negative; TN).

### Tissue harvesting

Immediately following the second MRI scan (seven days post injection), animals were pre-anesthetized via an intramuscular injection of ketamine-xylazine solution (20 and 2 mg/kg, respectively), terminally anesthetized by propofol overdose, and, after arrest of respiratory activity, they were transcardially perfused with 10% sucrose followed by 4% PBS-buffered paraformaldehyde (PFA). Perfusion pressure was maintained at 120–140 mmHg. Brains were harvested and post-fixed in 4% PFA for 48 h at 4°C. Then, brains were subdivided into 8–10 blocks, cryo-protected in 30% sucrose until sank, frozen on dry ice powder for five minutes and kept until cryo-sectioning.

### Post mortem analysis

Coronal sections 10 µm thick were cryo-sectioned on a Hyrax C25 PLMC cryostat (Zeiss, Warsaw, Poland) and processed for histology and immunohistochemistry. Sections from contralateral hemispheres were used as a control. For histological staining, eriochrome cyanine R (Merck Millipore, Billerica, MA, USA) and hematoxylin/eosin (both from Sigma) were used. For immunofluorescent staining, tissue sections were incubated with primary antibodies, mouse anti-neurofilament H (1:1000; Biolegend), rabbit anti-GFAP (1:500; Dako), and rabbit anti-Iba1 (1:500; Abcam), followed by secondary antibodies (Alexa Fluor 488 and 594; Life Technologies, Carlsbad, CA, USA; 1:500). Next, slices were counterstained with bis-benzimide H 33258 (Sigma) and mounted with FluoroGel (Electron Microscopy Sciences, Hatfield, PA, USA). The number of Iba-1 positive cells was calculated for six randomly selected ROIs.

### Image processing and statistical analysis

Images (both MRI and histopathology) were processed using ImageJ software (National Institutes of Health, Bethesda, Maryland, USA). Regions of interest for lesion segmentation were manually drawn in a blinded fashion. We performed standard validity analysis and calculated the positive predictive value (PPV) = TP/(TP/FP), negative predictive value (NPV) = TN/(TN/FN), sensitivity = = TP/(TP/FN) and specificity = TN/(FP/TN) for prediction of the injury area based on T1 gadolinium contrast [21]. The r Pearson was used for correlations. The PROC MIXED (SAS 9.4) has been used for remaining statistical analysis with the least square means used to detect difference between groups. A *p* value of less than 0.05 was considered statistically significant.

## Results

### MRI-guided CED infusion of gliotoxin

**Fig 2:**
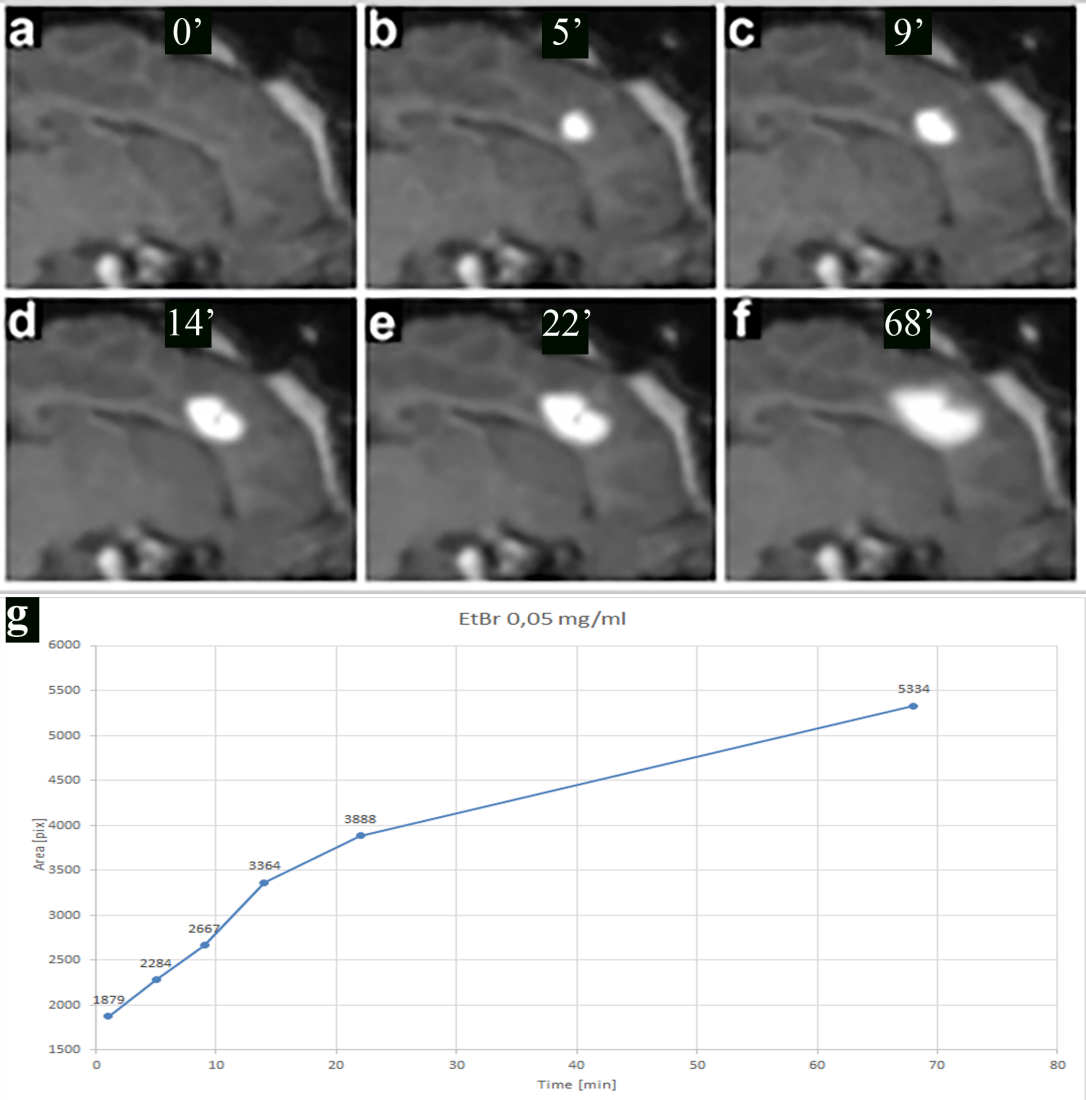
Convection-enhanced delivery of Gadolinium-supplemented gliotoxin. T1-weighted images were taken before (a), during (b-e) and directly after injection (f). Graph (g) is showing gliotoxin distribution over time during CED.

Using an MRI-compatible trajectory device and an injection cannula specifically designed to support convection-enhanced infusions (**Fig 1**), it was possible to reliably perform an intracerebral injection of toxin solution supplemented with Gadolinium. The entire procedure of gliotoxin targeting to the desired brain territory was monitored and guided on T1-weighted scans. While the catheter itself was not visible, T1-weighted images successfully visualized the brain parenchyma penetrated by injected solution in each of the injected animals. The area of hyperintensity on T1 images increased over one hour of the injection (**Fig 2**). There was neither leakage of gliotoxin outside the brain, nor hemorrhage observed. The contralateral hemisphere remained intact without any imaging abnormalities. In two animals, the initial placement of the injection cannula resulted in suboptimal biodistribution of gliotoxin outside the white matter but monitoring of that biodistribution in real-time facilitated quick identification of the problem and correction of the trajectory, further demonstrating the utility of the real-time MRI guidance (**S1 Fig**).

Overlap between intraoperative gliotoxin biodistribution vs. *post mortem* lesion size was assessed and there was a 100% overlap for EtBr (0.05 mg/ml) and a 74.56% overlap for LPC (30 mg/ml). On average, the overlap value was 76% for all eight animals. There was a strong (R = 0.66), but a statistically insignificant (p>0.05) correlation between gliotoxin biodistribution on the intraoperative T1 + Gd scan and hyperintensity size on T2 images at one week. Very strong correlation (R = 0.93, p<0.05) between the size of hyperintensity on T2 images and lesion area measured *post mortem* was confirmed.

### Analysis of the predictive value for real-time MRI of CED

Based on our calculations real-time guided CED of focal demyelination had following characteristics: PPV = 43±25%, NPV = 98±1%, sensitivity = 74.13±18% and specificity = 97±1%. This suggests that by using infusion of gadolinium and T1 MRI, we are able to predict at almost 43% certainty, the exact location of injury as detected by T2 MRI, and we are able to predict at above at 98% certainty which brain area will maintain intact. This method is further characterized by high sensitivity of 75%, and specificity 97%.

### Induction of lesion with ethidium bromide solution

**Fig 3:**
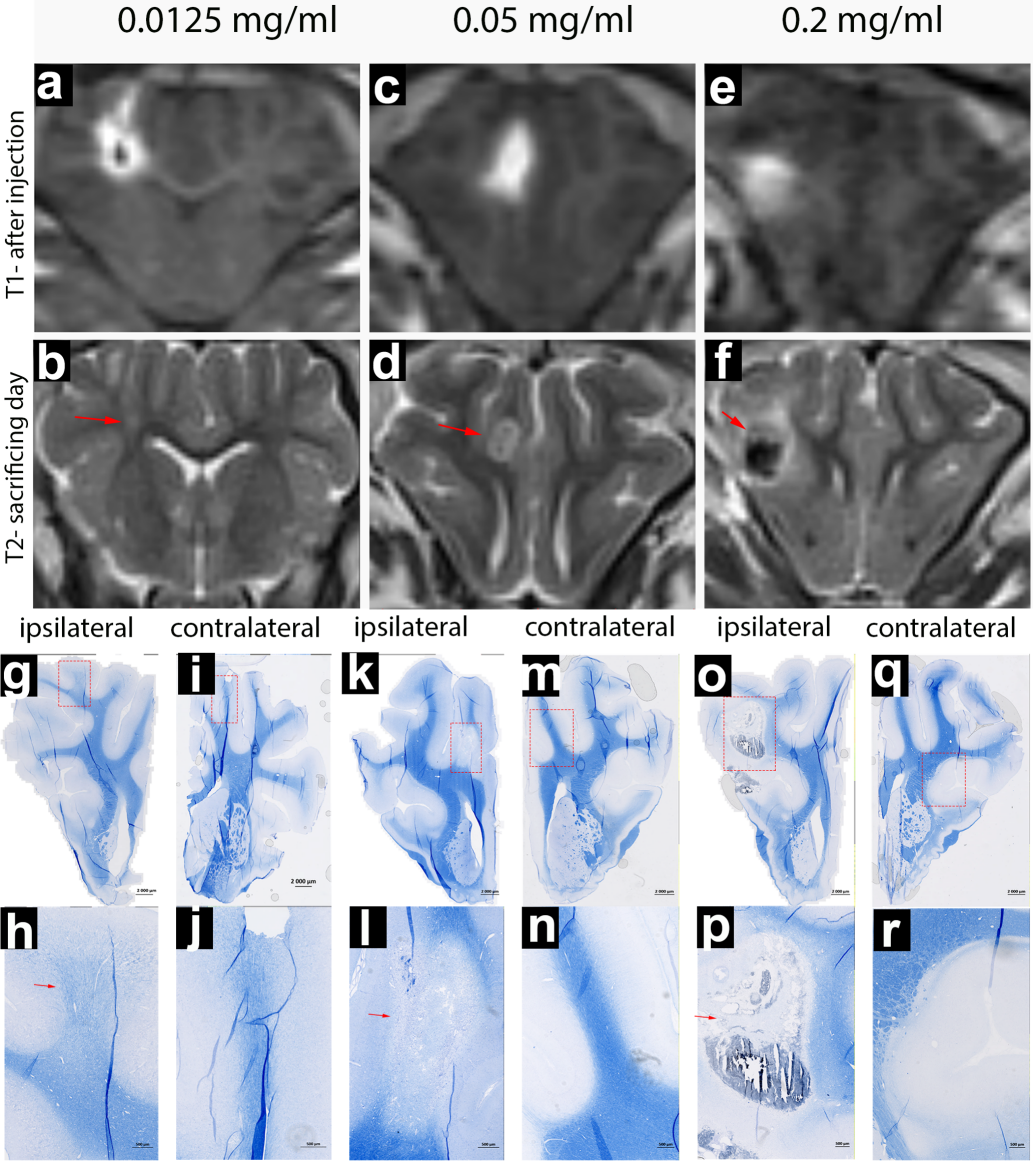
MR (a-f) and microscopic (g-r) images of lesioned brain treated with various doses of EtBr. MR images indicate hyperintense area directly after injection (a-e) and seven days later (b-f)) with red arrows indicating lesioned area. Eriochrome-stained coronal tissue sections from different doses of ethidium bromide were acquired as an overall view (g,j,k,m,o,q). Red rectangle marks lesion area that is shown at higher magnification (h,j,l,n,p,r). Red arrows indicate region with loss of myelin-specific blue color within the lesion.

**Fig 4:**
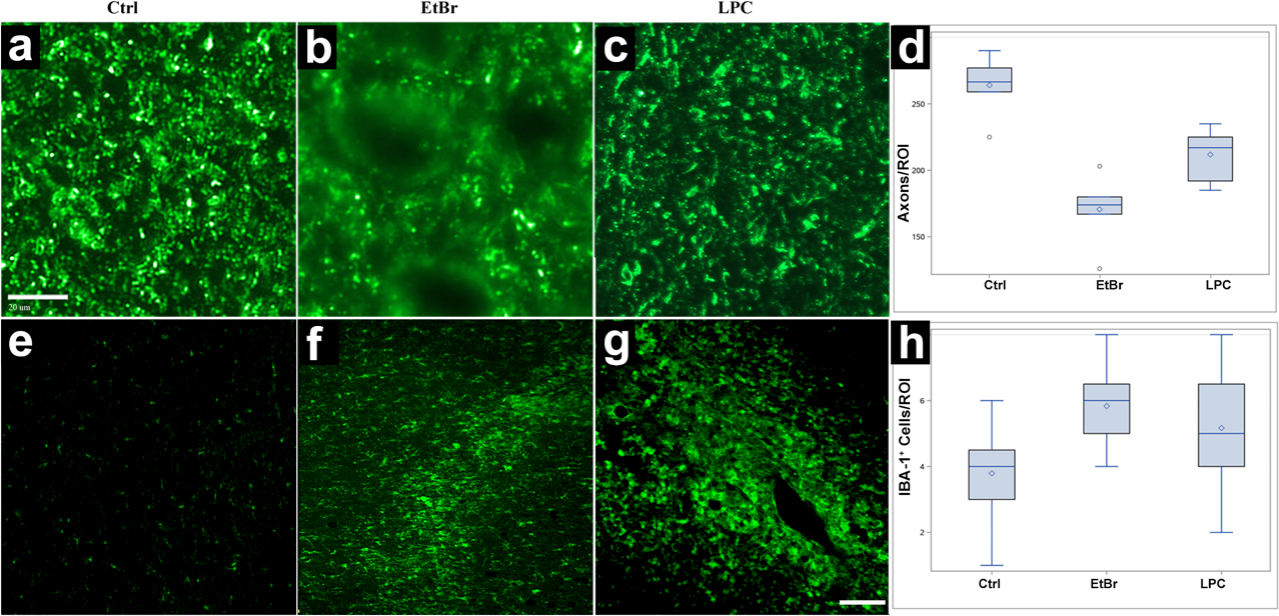
Immunohistochemistry of white matter damage induced with optimal doses of EtBr (0.05 mg/ml) or LPC (30 mg/ml), compared to normal non-injured white matter. Neurofilament staining (a-c) and staining for microglia specific IBA-1 (d-f) with comparison between groups for IBA-1-positive cells per ROI.

To investigate the optimal concentration of ethidium bromide (EtBr) for inducing focal demyelination, we performed injections with three different concentrations of this gliotoxin: 0.0125; 0.05; and 0.2 mg/ml. Imaging readouts included monitoring infusate distribution with T1+Gd scans during injection (**Fig 3A, 3C and 3E**) and lesion/hemorrhage assessment on T2-weighted MRI over a period of seven days. The 0.2 mg/ml EtBr resulted in evident vascular damage and hemorrhaging, as evidenced by hypointensity on T2w scans (**Fig 3F**). In contrast, the 0.0125 mg/ml dose had a minor effect, with subtle lesions on MRI (**Fig 3B**). Clearly visible demyelinating lesions without hemorrhage were observed following treatment with 0.05 mg/ml of toxin (**Fig 3D**). Histopathology with eriochrome staining for myelin was in good agreement with observations on MRI, with subtle lesions observed for the 0.0125 mg/ml dose (**Fig 3G–3J**), extensive demyelination when 0.05 mg/ml was used (**Fig 3K–3N**), and extensive tissue destruction for the 0.2 mg/ml dose (**Fig 3O–3R**). However, the 0.05 mg/ml dose of EtBr, resulted in decreased axonal density (**Fig 4B**) but evident immunoreactivity for neurofilaments in the lesion centers, indicating survival of demyelinated axons (**Fig 4B**). IBA-1 staining revealed there was activation of microglia as seen by increased intensity of green fluorescence (**Fig 4E and 4F**) and increased number of IBA-1-positive cells per ROI (**Fig 4G**).

### Induction of lesion with LPC solution

**Fig 5:**
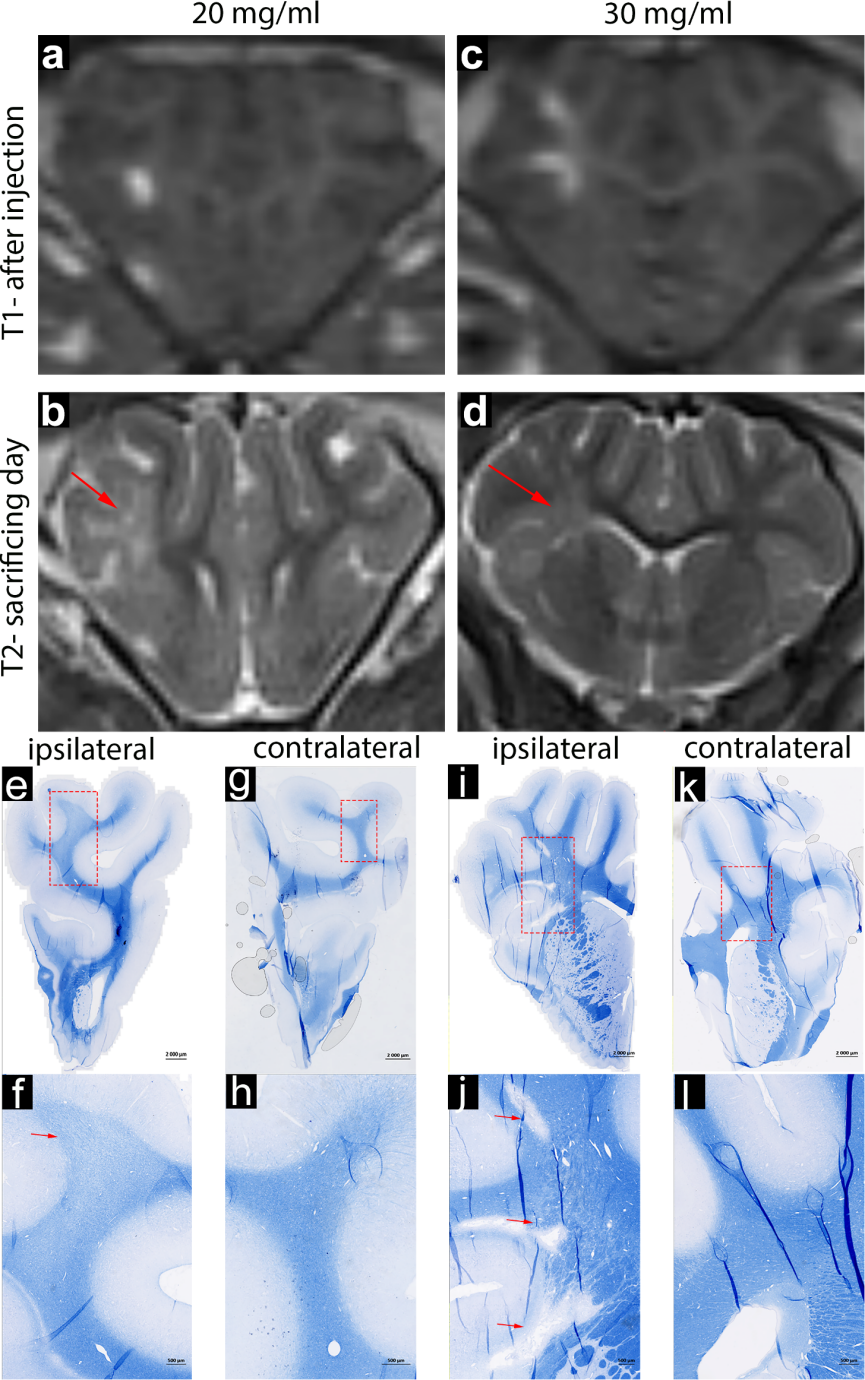
MR (a-d) and microscopic (e-l) images of lesioned brain treated with various doses of LPC. MR images indicate hyperintense area directly after injection (a,c) and seven days later (b,d); red arrows indicate lesioned area. Eriochrome-stained coronal tissue sections from different doses of lysolecithin were acquired as an overall view (e,g,i,k) and high magnification of the lesioned area in red box is shown below (f,h,j,l). Red arrows indicate loss of the myelin-specific blue color.

The impact of LPC on porcine white matter depends on the concentration. MRI and histopathological analysis indicated that treatment with both 20 and 30 mg/ml LPC resulted in hyperintensity on T2w MRI, indicating inflammatory process and/or demyelination (**Fig 5**); however, in histological eriochrome staining for myelin, 20 mg/ml LPC resulted in only a partial loss of myelin (**Fig 5E–5H**) and, at the 30 mg/ml dose, the lesions were more pronounced (**Fig 5I–5L**). Immunohistochemistry for IBA-1 revealed extensive activation of microglia in the 30 mg/ml treatment group (**Fig 4F and 4G**); importantly, there was only minor axonal loss, as evidenced by staining for neurofilaments (**Fig 4A and 4C**). For both 20 and 30 mg/ml treatment groups, there was a good preservation of tissue architecture, with no evidence of hemorrhage (**Figs 3 and 4**), which identifies LPC as less damaging gliotoxin.

### Comparison of using LPC and EtBr for lesion induction

We have observed more pronounced reduction in axon density for EtBr lesion compared to LPC lesions (170.67±25.15 vs. 211.83±19.37), while there was no difference in immune cell infiltration (5.83±1.19 vs. 5.16±1.64)). Interestingly, we have found that the area of T1 enhancement during CED procedure was larger for EtBr compared LPC injection, while there was no difference in the lesioned area between both toxins (p = 0.5).

## Discussion

To the best of our knowledge, this is the first study showing feasibility of induction of focal demyelination in large animal brain which would serve as a model of focal demyelination and remyelination. This is an example of directing efforts toward using animal models with better clinical relevance. The use of domestic pig as a model of human disease is increasing due to many beneficial factors, including the large size of the brain, the gyrencephalic structure, and the white matter/gray matter ratio that is much more similar to humans compared to that of rodents. The size, anatomic, and histological properties are superb advantages for using the pig in neurological disease modeling. While MS is typically a multifocal disease with unpredictable lesion distribution [22], this feature may be inconvenient when reproducibility is important for measuring therapeutic effect. If the white matter repair is pursued as therapeutic approach on preclinical stage, the utility of focal demyelination models of disease is favorable. Focal demyelination requires that the injection system precisely targets the area of interest. To precisely control the injection site and to obtain a focal demyelinating lesion, which was placed in the white matter, we selected a real-time, MRI-guided, convection-enhanced delivery approach (CED; [23]). This required a slow and stable infusion of toxin into the brain to enable the diffusion of the infused substance through the extracellular space [18]. The application of continuous pressure leads to the propagation of the toxin further into the brain parenchyma without damage. This method has been used in clinical trials as a delivery route for therapeutic agents [24]. CED enables precise demyelination of the area of interest with no reflux of the toxin along the injection cannula, which occurs with rapid infusion of large volumes [18]. The toxin solution was supplemented with gadolinium and using the MRI-compatible trajectory system facilitated real-time monitoring of the injection process. Constant monitoring helped us to target the corona radiata with great accuracy and in case of suboptimal delivery it was possible to immediately identify the problem and correct the placement of injection cannula. The high degree of T1 and T2 hyperintense area overlap further confirmed the successful prediction of lesion targeting. T1 hyperintensity to some extend overestimated lesion size as T2-weighted hyperintense area was smaller. Moreover, a comparison between the lesion area on MRI and histology suggested different diffusion rates of gliotoxin compared to what the contrast agent showed. One possible explanation of the imaging-histology mismatch is the toxin gradient within the injected area, with suboptimal concentration at the edge of the T1 + Gd hyperintensity. However, the difference in the diffusion rate between the contrast agent and the gliotoxin could also be a possible reason and this was observed earlier in an *in vivo* study [15]. Gadoteridol likely diffuses faster than both the toxins used in our study and this occurrence should be considered when the gadoteridol-enhanced CED procedure is performed. It has to be emphasized that abnormalities on T2 weighted MRI within the lesion reflects a combination of myelin loss and inflammatory processes.

We used two different gliotoxins, ethidium bromide (EtBr) and lysolecithin (LPC), due to their distinct demyelinating properties [19]. EtBr is a cytotoxic agent and depletion of myelin-forming cells after intracerebral injection was observed [25], whereas LPC acts as a myelin-solubilizing agent [25].

Injection of one of these toxins into the brain parenchyma induces focal demyelinating lesions, with relative sparing of axons in small animals [9]. The lesion induced by the EtBr solution is characterized by damage to all nucleated cells in the vicinity of stereotaxic injection, including astrocytes, oligodendrocytes, and even oligodendrocyte progenitors [25].

Another widely used toxin in focal demyelination studies is LPC, a molecule known as a detergent, as well as an activator of phospholipase A2 [25]. Injection of LPC into the CNS tissue results in myelin depletion, with relative sparing of the axons [9, 26]. Therefore, focal toxin-induced demyelination is a valuable model for remyelination studies.

Our results indicate that 30 mg/ml of LPC and 0.05 mg/ml of EtBr are optimal concentrations of toxin for CED infusion in the 30-kg juvenile pig brain. Our histopathological examination has shown demyelination spatially corresponding to that observed in MRI. Histopathological features of demyelinating lesions included loss of myelin, reduction of axonal density and microglial activation showing relevance to clinically observed demyelinating lesions [27]. Experiments in rat cerebellum cultures indicated that LPC can produce rapid demyelination, while studies with brain tissue homogenate have shown that LPC can solubilize brain myelin [28].

The infusion of 0.0125 mg/ml EtBr and 20 mg/ml LPC resulted in insufficient loss of myelin as visualized by both T2w MRI and eriochrome staining. Immunofluorescent staining confirmed the suboptimal concentration of the toxic agent, as a loss of myelin was not observed. Furthermore, injection of 0.2 mg/ml EtBr resulted in severe demyelination, hemorrhage, and a high degree of tissue damage. In contrast, the lesioned areas after the injection of 30 mg/ml LPC or 0.05 mg/ml EtBr were full of cellular infiltrates, with mild astrocytosis and loss of myelin density that corresponded with the histopathology in EAE [9]. For 30 mg/ml LPC and 0.05 mg/ml EtBr, axonal degeneration was not observed on immunofluorescent staining. Moreover, tissue was free of hemorrhages and direct cytotoxic damage. Hyperintensity was evident on T2-weighted images which indicates both demyelination and inflammatory processes at this acute stage and that is reflecting active demyelinating lesions found in MS patients [29] and the lack of compact myelin observed in hypomyelinated mice [28]. The loss of blue color in eriochrome staining indicated demyelination, which corresponds to the pattern of myelin loss observed in hypomyelinated mice [5]. Therefore, when all experimental settings were compared, the histopathological outcome from 30 mg/ml LPC and 0.05 mg/ml EtBr appeared to be optimal for inducing focal demyelination. Limitation of this study was relatively short follow-up period not allowing for assessment of spontaneous remyelination.

## Conclusions

Convection-enhanced delivery, in concert with real-time MRI guidance, is an excellent tool to induce focal demyelinating lesions in pigs. We found that ethidium bromide at the concentration of 0.05 mg/ml) and lysolecithin at the concentration of 30 mg/ml) are effective for inducing focal demyelination in the porcine brain. Furthermore, lesion evolution could be monitored using both T1 and T2-weighted MRI and validated by histopathology. The LPC resulted with better preservation of tissue cyto-architecture and less intensive inflammation, which may be a preferred model for a first-line testing of re-myelination therapies. This model may become very useful for testing novel therapeutic strategies, including those based on stem cell transplantation.

## Supporting information

S1 Fig Real-time MRI of gliotoxin injection is showing misplaced cannula (a). after repositioning of the injection cannula gliotoxin is delivered precisely to the target (b).
(TIF)
