# Supplementary figures and images for: MRI-guided intracerebral convection-enhanced injection of gliotoxins to induce focal demyelination in swine
Source: PLoS One. 2018 Oct 1;13(10):e0204650. doi: 10.1371/journal.pone.0204650 (PMC6166947; doi:10.1371/journal.pone.0204650)

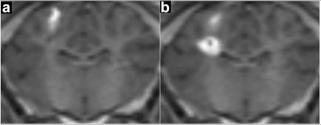

Supplement: S1 Fig — Real-time MRI of gliotoxin injection is showing misplaced cannula (a). after repositioning of the injection cannula gliotoxin is delivered precisely to the target (b). (TIF) [file pone.0204650.s001.tif]
